# miR-181c-5p/DERL1 pathway controls breast cancer progression mediated by TRAF6-linked K63 ubiquitination of AKT

**DOI:** 10.1186/s12935-024-03395-1

**Published:** 2024-06-10

**Authors:** Yang Bai, Zhanqiang Zhang, Jiong Bi, Qian Tang, Keying Jiang, Chen Yao, Wenjian Wang

**Affiliations:** 1 Laboratory of Department of Surgery, the First Affiliated Hospital of Sun Yat-Sen University, Guangzhou, 510080 Guangdong China; 2 Department of Thyroid, the First Affiliated Hospital of Sun Yat-Sen University, Guangzhou, 510080 Guangdong China; 3 Department of Anesthesiology, Guiqian International General Hospital, Guiyang, 550000 Guizhou China

**Keywords:** DERL1, Breast cancer, Prognosis, K63-ubiquitination, Micro RNA

## Abstract

**Background:**

Aberrant Derlin-1 (DERL1) expression is associated with an overactivation of p-AKT, whose involvement in breast cancer (BRCA) development has been widely speculated. However, the precise mechanism that links DERL1 expression and AKT activation is less well-studied.

**Methods:**

Bioinformatic analyses hold a promising approach by which to detect genes’ expression levels and their association with disease prognoses in patients. In the present work, a dual-luciferase assay was employed to investigate the relationship between DERL1 expression and the candidate miRNA by both in vitro and in vivo methods. Further in-depth studies involving immunoprecipitation-mass spectrum (IP-MS), co-immunoprecipitation (Co-IP), as well as Zdock prediction were performed.

**Results:**

Overexpression of DERL1 was detected in all phenotypes of BRCA, and its knockdown showed an inhibitory effect on BRCA cells both in vitro and in vivo. The Cancer Genome Atlas (TCGA) database reported that DERL1 overexpression was correlated with poor overall survival in BRCA cases, and so the quantification of DERL1 expression could be a potential marker for the clinical diagnosis of BRCA. On the other hand, miR-181c-5p was downregulated in BRCA, suggesting that its overexpression could be a potent therapeutic route to improve the overall survival of BRCA cases. Prior bioinformatic analyses indicated a somewhat positive correlation between DERL1 and TRAF6 as well as between TRAF6 and AKT, but not between miR-181c-5p and DERL1. In retrospect, DERL1 overexpression promoted p-AKT activation through K63 ubiquitination. DERL1 was believed to directly interact with the E3 ligase TRAF6. As Tyr77Ala or Tyr77Ala/Gln81Ala/Arg85Ala/Val158Ala attempts to prevent the interaction between DERL1 and TRAF domain of TRAF6, resulted in a significant reduction in K63-ubiquitinated p-AKT production. However, mutations in Gln81Ala, Arg85Ala, or Val158Ala could possibly interrupt with these processes.

**Conclusions:**

Our data confirm that mediation of the miR-181c-5p/DERL1 pathway by TRAF6-linked AKT K63 ubiquitination holds one of the clues to set our focus on toward meeting the therapeutic goals of BRCA.

**Supplementary Information:**

The online version contains supplementary material available at 10.1186/s12935-024-03395-1.

## Highlights

Evidence of overexpression of DERL1 in several subtypes of BRCA suggests its oncogenic role.

Mediation of miR-181c-5p with DERL1 suppresses BRCA progression.

DERL1 promotes AKT activation through AKT K63 ubiquitination.

DERL1 binds with such TRAF6 (a cytosol E3 ligase) residues as TYR-77, GLN-81, ARG-85, and VAL-158.

Mediation of miR-181c-5p/DERL1 pathway by TRAF6-linked AKT K63 ubiquitination modulated BRCA progression.

**Supplementary Information:**

The online version contains supplementary material available at 10.1186/s12935-024-03395-1.

## Introduction

Breast cancer (BRCA) is a complex and heterogeneous malignant disease encompassing multiple genes and/or signal pathways [1–3]. However, much less information is available regarding the spectra of genes supposed to be associated with BRCA prognosis. To some extent, this might explain the limited therapeutic choices addressing BRCA and the restrained hope for a disease-free state among patients and treating physicians. Currently, BRCA accounts for 15.5% of all cancer-relevant deaths [4]. Thus, the identification of more critical genes and their signal pathways involved is a panacea to raise new hopes for improving BRCA treatment [5].

DERL1, an endoplasmic reticulum (ER) membrane protein, has been widely overexpressed in human cancers due to various factors inducing ER stress [6–8]. DERL1 was first identified as a causative agent in ER-associated degradation (ERAD) [9–11]. It also plays a key role in ubiquitinated protein degradation of cytosol [12]. Human DERL1 binds with p97/VCP/ubiquitin ligases and forms a transmembrane homotetramer tunnel to translocate the misfolded proteins across the ER membrane [13]. Many of the key ERAD clients not only have a role in taking out the misfolded proteins from ER but are also involved in modulating ER’s biological pathways [14]. However, little is known whether DERL1 is associated with ubiquitinated protein modification besides ubiquitinated protein degradation.

MicroRNAs (miRNAs) are a class of master regulators controlling more than 60% of human protein-coding gene transcriptions, and their aberrant expressions are closely associated with tumorigenesis [15, 16]. This is why cancer drugs targeting miRNAs receive significant attention from practitioners [17]. miR-181c-5p is believed to act as a suppressor or an oncogene in different cancers [18–20]. In a study on oral squamous cell carcinoma, melatonin-induced miR-181c-5p downregulated DERL1 [21]. Whether DERL1 overexpression could be directly regulated by miR-181c-5p in BRCA cells has not been investigated so far.

In mammalian cells, tumor necrosis factor receptor-associated factor 6 (TRAF6) is one among the family of TRAF proteins (TRAF1–TRAF7), a class of cytoplasmic adaptors [22]. TRAF6 has a RING domain at the N-terminus with E3 ubiquitin ligase activity and a TRAF domain at the C-terminus for interacting with the cytoplasmic domain of receptors [23, 24]. AKT (also called protein kinase B), which is synthesized in the ER, is said to play a central role in regulating cancer cells [25, 26]. K63-linked ubiquitination is a key step in AKT activation [27]. To that end, TRAF6, a direct E3 ligase of AKT, is thought to be critical for K63-linked ubiquitination of AKT [28]. However, the mechanism of TRAF6-mediated K63 ubiquitination of AKT is obscure. On the other hand, there is growing evidence suggesting DERL1 could promote tumor development via the activation of AKT [29, 30]. Whether DERL1 promotes K63-ubiquitinated AKT activation in a process mediated by TRAF6 is not known.

In the present study, overexpression of DERL1 is implicated in all subtypes of BRCA based on molecular studies of cell lines coupled with database analyses. Thus, overexpression of DERL1 stands as an independent prognostic factor for predicting poor overall survival (OS) in BRCA patients. Moreover, DERL1 substantially promoted BRCA cells’ malignant phenotypes. Our data showed that miR-181c-5p directly inhibited DERL1 protein expression. The upregulation of miR-181c-5p suppressed DERL1’s oncogenic role, which would favor the therapeutic outcomes for BRCA patients. DERL1 stimulated p-AKT production via the K63-ubiquitinated AKT pathway. Notably, our study evidenced a direct interaction of DERL1 with TRAF6. Moreover, overexpression of DERL1 promoted AKT phosphorylation via TRAF6-mediated K63-linked polyubiquitination of AKT. TYR-77, GLN-81, ARG-85, and VAL-158 residues of DERL1 might be involved in its interaction with the C-terminus of TRAF6.

## Materials and methods

### Data source and preprocessing

RNAseq data and corresponding clinical information of 1,226 BRCA patients were downloaded from the Cancer Genome Atlas (TCGA). After discarding records lacking supporting clinical information, 111 normal and 1,069 tumor tissue RNAseq data with clinical information at level 3 HTSeq-FPKM were converted into TPM (transcripts per million reads) format for further analysis. Unavailable or unknown clinical information was considered as missing values. Based on the expression levels of DERL1, tumor samples were divided into low- and high-expression groups.

### Cell culture

One normal breast epithelial cell line (MCF10A) and eight BRCA cell lines (ZR-7530, AU565, MCF-7, SKBR-3, BT-474, T-47D, MDA-MB-231, and MDA-MB-468) were obtained from the American Type Culture Collection (ATCC). All cells were cultured in respective media supplemented with 1% penicillin/streptomycin (Gibco/Thermo Fisher Scientific) and 10% fetal bovine serum (Gibco) in a 5% CO_2_ cell incubator at 37 °C.

### RNA interference, overexpression, and cell transfection

DERL1, TRAF6, AKT, and Ubiquitin-overexpressing plasmids, siRNAs, and their corresponding negative controls were purchased from HanBio. shRNA sequences targeting DERL1 were designed based on siDERL1-1. MiR-181 C-5p mimic, NC mimic, hsa-miR-181c-5p primer, and U6 primer were purchased from RiboBio (Supplementary materials). During transfection, Lipofectamine-3000 (Invitrogen) was used to transfect plasmids per the manufacturer’s instructions, while RNAiMAX (Invitrogen) was used to transfect siRNAs and mimics. Subsequently, cells were collected for RNA extraction at 24–48 h post-transfection, and for protein extraction at 48–72 h post-transfection. In addition, sh-DERL1 and sh-NC as a lentiviral system (Hanbio) coupled with a puromycin selection marker were stably transfected into the cells.

### Quantitative real-time PCR

TRIZOL (Thermo) was used to extract total RNA from the cells according to standard protocol. A NanoDrop 2000 spectrophotometer (Thermo) was used to measure RNA concentration, which was then quantified by quantitative real-time PCR (qRT-PCR) using a qPCR premix (AG). CT values were exported from the exponential phase of PCR directly into a worksheet for further analysis. The primers used are shown in Supplementary materials. Gene expression levels were calculated relative to the housekeeping gene GAPDH.

### Immunoprecipitation of protein blots

Treated cells were washed with PBS and lysed in RIPA lysis buffer (Beyotime) supplemented with a protease inhibitor cocktail (Beyotime) to extract total proteins. Membrane proteins were extracted from the cells using a Membrane Protein Extraction Kit (Beyotime). MG132, a proteasome inhibitor, was employed to inhibit degradation of ubiquitinated proteins. Thus, all experimental cells were treated with 10 µM MG132 (Medchemexpress) for 4 h prior to lysis. Protein concentration was determined using the BCA assay. After separation using SDS-PAGE, proteins were transferred to polyvinylidene difluoride (PVDF) membranes (Thermo Fisher Scientific), which were incubated with 5% BSA for 1 h and then treated with primary antibodies overnight at 4 °C. After washing three times with TBST and incubation with secondary antibodies, the signal intensity of protein complexes was detected using an enhanced chemiluminescence reagent (Millipore).

Co-immunoprecipitation (Co-IP) was performed as follows. Cells were lysed in a cold lysis buffer (50 mM Tris-Cl at pH 7.4, 150 mM NaCl, 1 mM EDTA, 1% NP-40, 0.25% sodium deoxycholate, protease inhibitor mixture). Subsequently, cell extracts (500 µg) were incubated with primary antibodies or control IgG on a rotator overnight at 4 °C, followed by the addition of protein G magnetic beads (Invitrogen) for 2 h at 4 °C. The beads were then washed four times using a lysis buffer and immune complexes subjected to SDS-PAGE prior to analysis by immunoblotting using a secondary antibody. Details of the antibodies are shown in Supplementary materials.

### Protein complex identification by LC-MS/MS

Transfection of HA-DERL1 was performed on ZR-7530 and MDA-MB-231 cells, and HA-binding magnetic beads were used to immunoprecipitate HA-tagged DERL1 protein and its complexes. After chromatographic separation (Easy-nLC 1000, Thermo Fisher) of protein complexes, LC-MS/MS analysis was conducted using a Q Exactive mass spectrometer (Thermo Fisher). The detection mode was positive ionization, with a mass range of 300–1800 m/z. The resolution of first-level mass spectrometry was set at 70,000 at 200 m/z, with an automatic gain control (AGC) target of 1e6 and a maximum ion trap (IT) of 50 ms. Dynamic exclusion time was set to 30.0 s. Peptide and peptide fragment mass-to-charge ratios were computed as follows: after each full scan, 20 fragment spectra (MS2 scans) were acquired. The chosen type of MS2 activation was higher-energy collisional dissociation (HCD), with an isolation window of 2 m/z. The resolution of second-level mass spectrometry was set at 17,500 at 200 m/z, and the normalized collision energy was set to 27 eV. Underfill was set to 0.1%. For the analysis of raw files obtained from mass spectrometry, Proteome Discoverer 2.5 search engine was used to search against the UniProt database (www.uniprot.org) in order to identify the proteins.

### Dual-luciferase assay

WT and mutant oligonucleotides containing the 3’-UTR region with the miR-181 C-5p-binding site, or the promoter region with the DERL1-binding site, were cloned into the luciferase plasmid. Subsequently, the luciferase reporter gene was transfected into 293T cells along with the miR-181 C-5p mimic or DERL1 plasmid and its negative control following published protocol. Luciferase activity was measured using a dual-luciferase reporter kit (Transgene) according to manufacturer’s instructions.

#### Wound-healing assay

Transfected BRCA cells were resuspended into six-well plates. Once the cells reached over 90% confluence, scratches were created on cell surface using a 200 µl pipette tip. Subsequently, the cells were cultured in a DMEM medium supplemented with 2% FBS. The scratch areas were observed at 0 and 24 h using a DMi 8 microscope (Leica).

### Transwell assay

For this purpose, a Transwell chamber with an 8 μm filter (Corning) was used. For migration assays, 20,000 cells per well were seeded into the upper chamber in a serum-free medium, and for invasion assays, the upper chamber was precoated with Matrigel (BD Science). A complete medium containing 10% FBS was added to the lower chamber.

### Cell proliferation assay

Cell proliferation assay was performed using EdU assay kit (Beyotime) according to manufacturer’s guidelines. After transfection, ZR-7530 and MDA-MB-231 cells (5,000 cells per well) were plated into 96-well plates and incubated with 10 µM EdU for 2 h at 37℃. Cells were then fixed with 4% paraformaldehyde for 30 min, permeabilized with 0.5% Triton X-100 for 15 min, treated with a staining reagent for 30 min, stained with DAPI for nearly 10 min, and then visualized under a DMi 8 microscope (Leica).

### Apoptosis assay

Apoptosis was analyzed by flow cytometry using the Annexin V-FITC Cell Apoptosis Kit (Beyotime) according to manufacturer’s instructions. Within 48 h after transfection, cells were harvested, suspended in a binding buffer, and then stained with fluorescein isothiocyanate (Annexin V) and propidium iodide (PI) for 15 min at room temperature in the dark. Finally, apoptosis rates were measured using CytoFLEX (Beckman Coulter).

### Immunofluorescence cell staining

ZR-7530 and MDA-MB-231 cells cultured on sterile glass coverslips were washed three times with pre-cooled PBS, fixed with 4% paraformaldehyde for 10 min, incubated with 0.2% Triton X-100 solution for 10 min, and then treated with 10% BSA blocking solution for 30 min at room temperature. Cells were incubated overnight with anti-DERL1 or TRAF6 antibodies at 4℃ and then with fluorescent secondary antibodies (anti-mouse IgG2a Alexa Fluor 488 and anti-rabbit Alexa Fluor 594) for 1 h. Finally, cells were washed with PBS and nuclei were stained with DAPI for 5 min at room temperature. Immunofluorescence was assessed under a DMi 8 microscope (Leica).

### Experimental animals

The animal protocol was approved by the Animal Ethics Committee of the First Affiliated Hospital of Sun Yat-sen University. Four-week-old female BALB/c nude mice (purchased from the Experimental Animal Center of the First Affiliated Hospital of Sun Yat-sen University) were used in experiments. Lentivirus sh-DERL1 and sh-NC (9 × 10^8^ TU/ml, Hanbio) were used to infect the MDA-MB-231 cell line, achieving a stable knockdown version of DERL1 cell line. A subcutaneous tumor model (2 × 10^6^ cells in PBS) and a distant metastasis model (5 × 10^6^ cells in PBS) were created by tail vein injection. All experimental mice were housed in the SPF animal facility at the Experimental Animal Center. After 40 days, the nude mice in the subcutaneous tumor-forming group were euthanized, and tumor size was measured (V = 1/2 × length of tumor × width^2^). Forty days later, mice in the distant metastasis group were imaged using an in vivo imaging system and then euthanized by cervical dislocation. Lung tissues were harvested, subjected to HE staining, and photographed to study tumor formation and metastatic lesions.

### Protein-protein docking

The structures of DERL1 and TRAF6 were obtained by accessing the Protein Data Bank (https://www.rcsb.org/). Before proceeding with docking, pre-processing steps under the Chemical Computing Group Molecular Operating Environment included preparing the protein structures, performing protonation, assigning partial charges, and refining the energy of the system. For actual protein-protein docking, the Zdock server (https://zdock.umassmed.edu/) was utilized, and for the plotting of results, PyMOL 2.5 (https://pymol.org/2/) was used.

### Statistical analysis

SPSS Statistics 22.0 and GraphPad Prism 9 were used for statistical analyses of data. The Mann-Whitney U rank sum test was used to assess differences in the expressions of genes between normal tissues and BRCA tissues. For correlation analysis, Spearman’s correlation test was used. Student’s t-test and one-way ANOVA were used to detect differences between two or more groups. A *P* value < 0.05 signified statistical significance.

## Results

### DERL1 is overexpressed in BRCA subtypes and is an independent predictor of patients’ overall survival

**Fig. 1:**
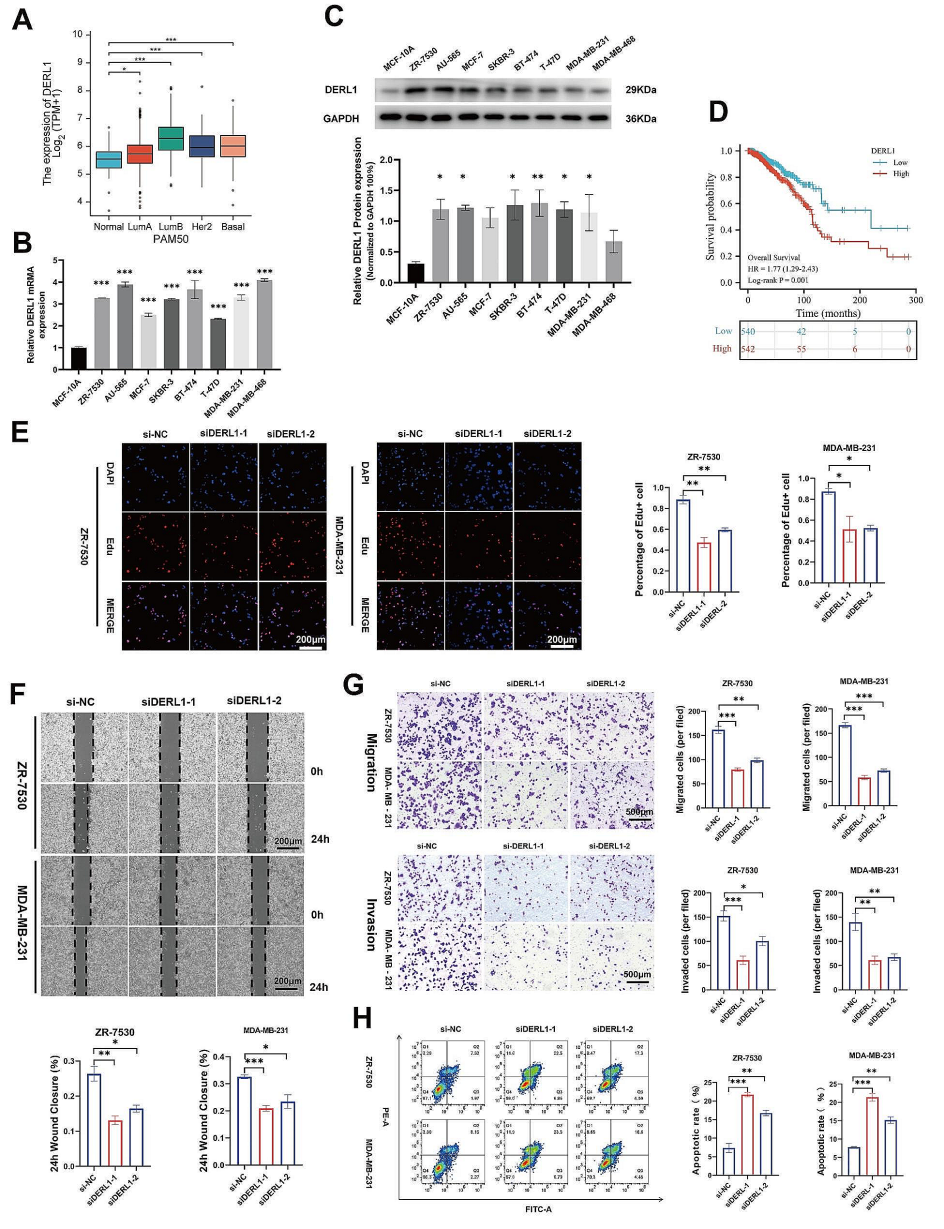
Expression of DERL1 and its oncogenic action in BRCA. **A.** Expression of DERL1 in different subtypes of BRCA based on TCGA data. **B.** Relative mRNA expression of DERL1 in a BRCA cell line compared to a normal breast cell line. **C**. Expression of DERL1 protein in normal breast cell line and BRCA cell line as detected by immunoblot. **D.** Overall survival (OS) curves of DERL1-high vs. DERL1-low patients using TCGA data. **E.** EdU assay to assess the viability of ZR-7530 and MDA-MB-231 cells after transfection with si-DERL1-1, si-DERL1-2, or si-NC. Scale bar, 200 μm. **F.** Evaluation of mobility in DERL1-depleted ZR-7530 and MDA-MB-231 cells using a wound-healing assay. Scale bar, 200 μm. **G.** Assessment of migration and invasiveness of ZR-7530 and MDA-MB-231 cells after DERL1 deletion using a Transwell assay. Scale bar, 500 μm. **H.** Impact of DERL1 on apoptosis of ZR-7530 and MDA-MB-231 cells. Data are presented as mean ± SEM (*n* = 3), *P < 0.05; **P < 0.01; ***P < 0.001

Using the TIMER database (https://cistrome.shinyapps.io/timer/), the differential expression of DERL1 between various tumors and normal tissues was evaluated, which suggested an upregulation in 11 different tumors, including BRCA, while a downregulation in three tumors (Fig. S1A), consistent with previous reports [6, 7]. Furthermore, utilizing the TCGA database (https://portal.gdc.cancer.gov), a significant overexpression of DERL1 mRNA was found in both unpaired and paired BRCA samples (Fig. S1B-C) and across all subtypes of BRCA (Fig. 1A). This observation was further validated by qPCR (Fig. 1B) and immunoblot (Fig. 1C) in BRCA cell lines.

Next, using the TCGA database, we investigated the impact of DERL1 on the prognoses of BRCA patients. Based on the log-rank test, patients with low DERL1 expression had significantly longer OS (Fig. 1D) and long-term disease-specific survival (DSS) (Fig. S1D) than those with high DERL1 expression. Univariate and multivariate regression analyses demonstrated that DERL1 expression serves as an independent prognostic predictor of OS (Table 1). A Cox regression model was constructed based on age (< 60 or 60), lymph node status (N0 or N1-3), M stage (M0 or M1), and DERL1 expression (high or low). The total score obtained from summing the assigned points for each factor in the nomogram indicated that higher scores represented worse outcomes (Fig. S1E). The prognostic calibration plot, used for fitting the Cox regression model, showed that the bias correction line closely approximated the ideal curve (the 45-degree line), indicating good agreement between the predicted and observed values for 1-, 3-, and 5-year survival (Fig. S1F).

**Table 1:** Univariate and multivariate regression analysis of invasive breast cancer based on the TCGA database

Characteristics	Univariate analysis			Multivariate analysis	
	Hazard ratio (95% CI)	*P* value		Hazard ratio (95% CI)	*P* value
T stage (T3&T4 vs. T1&T2)	1.608 (1.110–2.329)	0.012		1.365 (0.905–2.060)	0.138
N stage (N1&N2&N3 vs. N0)	2.239 (1.567–3.199)	< 0.001		2.062 (1.396–3.046)	< 0.001
M stage (M1 vs. M0)	4.254 (2.468–7.334)	< 0.001		2.171 (1.184–3.980)	0.012
Age (> 60 vs. < = 60)	2.020 (1.465–2.784)	< 0.001		2.156 (1.514–3.070)	< 0.001
PAM50 (basal vs. LumA&LumB&Her2)	1.040 (0.695–1.558)	0.848			
PR status (positive vs. negative)	0.732 (0.523–1.024)	0.068			
ER status (positive vs. negative)	0.712 (0.495–1.023)	0.066			
HER2 status (positive vs. negative)	1.593 (0.973–2.609)	0.064			
DERL1 (high vs. low)	1.775 (1.271–2.479)	< 0.001		1.574 (1.093–2.265)	0.015

### DERL1 functions as an oncogene

Immunofluorescence and immunoblot analyses were performed to examine the localization of DERL1 in BRCA cells, which demonstrated that DERL1 predominantly localized to the plasma membrane of the cells, which is consistent with literature [9] (Fig. S2A-B).

To investigate the impact of DERL1 on BRCA cell phenotypes, we utilized ZR-7530 and MDA-MB-231 cell lines. Edu assays revealed that the knockdown of DERL1 significantly inhibited cell proliferation (Fig. 1E, Fig. S2C-D). Wound healing and Transwell assays demonstrated that migration and invasion abilities of BRCA cells were suppressed upon DERL1 silencing (Fig. 1F-G). Moreover, the knockdown of DERL1 increased the proportion of apoptotic cells (Fig. 1H). These findings suggest that DERL1 is crucial for the growth of BRCA cells.

**Fig. 2:**
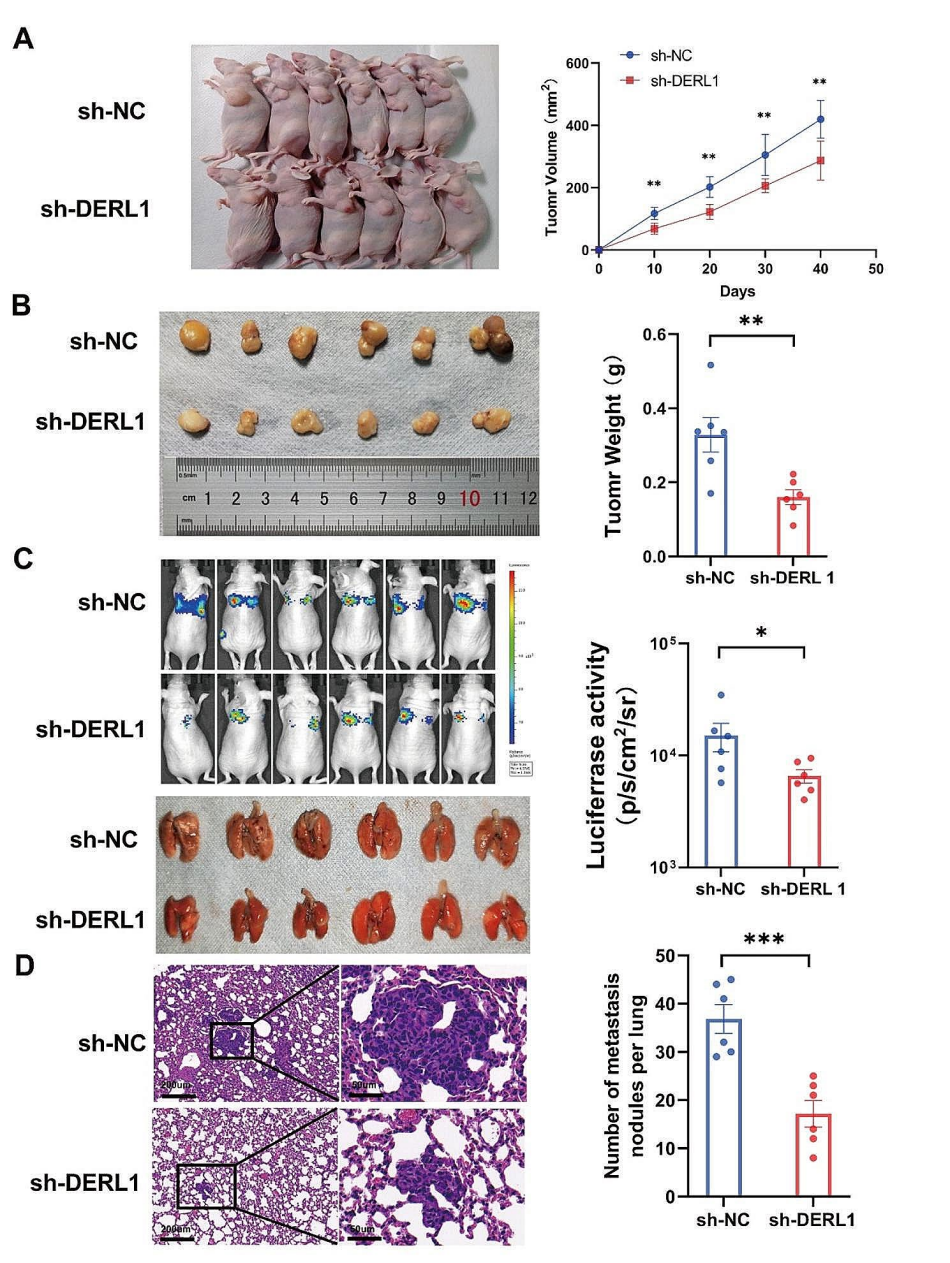
Overexpression of DERL1 promotes the growth and metastasis of BRCA cells *in vivo*. **A-B.** MDA-MB-231 cells treated with sh-NC or sh-DERL1 were subcutaneously injected into all experimental mice. On every 10th day, tumor volume in each mouse was estimated using the formula: (shortest diameter)² × (longest diameter) × 0.5. Tumor weight in each mouse was evaluated on day 40 after euthanization. **C.** Bioluminescence imaging of lung metastases in nude mice was performed on day 40 after injecting them with D-luciferin. **D.** Mice were sacrificed on day 40. H&E staining of metastatic lung tissues was performed and the observations recorded followed by statistical analyses. *N* = 6 mice in each in vivo group. Data are presented as mean ± SEM (*n* = 3), *P < 0.05; **P < 0.01; ***P < 0.001

In an in vivo subcutaneous xenograft model, depletion of DERL1 inhibited tumor growth in MDA-MB-231 cells (Fig. 2A-B). To further study the effect of DERL1 on tumor metastasis, MDA-MB-231-luc cells stably transfected with DERL1 were injected into nude mice via the tail vein. On day 40, lung fluorescence intensity was measured via bioluminescence imaging after an intraperitoneal injection of 100 µl 15 µg/µl D-luciferin (Fig. 2C). In addition, the number of cross-section nodules in lung tissues were recorded after staining with hematoxylin and eosin (HE) (Fig. 2D). Depletion of DERL1 was evidenced by a reduction in fluorescence intensity and a decrease in the number of nodules. Put together, our findings suggest a significant oncogenic role for DERL1 in BRCA development, consistent with literature [6, 30].

### DERL1 promotes AKT activation via the K63-linked ubiquitination pathway in BRCA

**Fig. 3:**
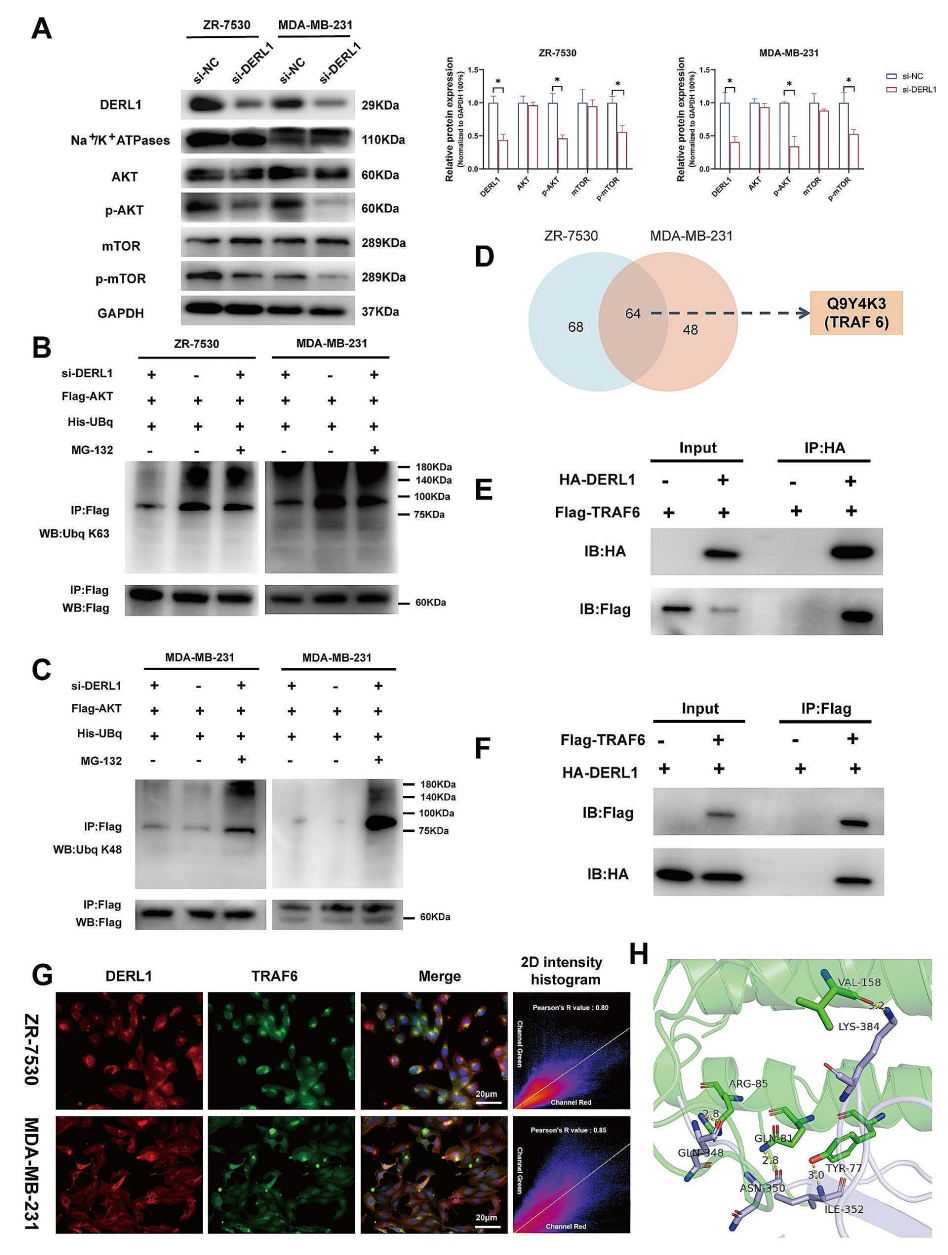
Mechanism by which DERL1 regulates AKT activation. **A.** Protein levels associated with the AKT signaling pathway were assessed by immunoblot in the context of DERL1 overexpression. **B-C.** ZR-7530 and MDA-MB-231 cell lysates were immunoprecipitated with anti-Flag antibodies and immunoblotted for K63-linked ubiquitination and AKT **(B)** or K48-linked ubiquitination and AKT **(C)**. **D.** Sixty-four DERL1-interacting proteins were identified in ZR-7530 (blue) and MDA-MB-231 (red) cells – TRAF6 being one among them. **E-F.** Expression of Flag-DERL1 and HA-TRAF6 in HEK-293T cells. The HA-tagged TRAF6 was immunoprecipitated and subjected to immunoblotting (IB) using an HA antibody. Similarly, Flag-tagged DERL1 was immunoprecipitated and subjected to IB using a Flag antibody. **G.** Immunofluorescence of ZR-7530 and MDA-MB-231 cells using an anti-DERL1 monoclonal antibody (red) and an anti-TRAF6 polyclonal antibody (green). After staining with DAPI, nuclear images were captured at a magnification of ×200 with 1× digital zoom, and scale bars represent 100 μm. **H.** Image shows strong hydrogen bonding between TYR-77, GLN-81, ARG-85, and VAL-158 in DERL1 (green) and ILE-352, ASN-350, GLU-348, and LYS-384 in TRAF6 (grey), respectively, with a bond length of 3.0 Å, 2.8 Å, 2.8 Å, and 3.2 Å, suggesting that these residues may be the active ones. *P < 0.05; **P < 0.01; ***P < 0.001; MG132: a potent, reversible proteasome inhibitor

Evidence linking the association between DERL1 overexpression and enhanced AKT activity and subsequently the latter’s role in tumorigenesis is well reported [29, 30]. We individually knocked down DERL1 in ZR-7530 and MDA-MB-231 cell lines and observed a significant reduction in the protein levels of p-AKT and p-mTOR, but not of AKT and mTOR (Fig. 3A). Although the present evidence strongly links DERL1 overexpression with AKT activation, the specific signaling pathway through which DERL1 regulates AKT remains uncertain. Prior studies restricted their focus to DERL1 as a protein-conducting channel in the ERAD machinery for signaling proteasomal degradation [31].

It is now clear that besides K48-linked ubiquitination, K63-linked polyubiquitination too seems to be a critical step in AKT activation [32]. GSEA analysis revealed DERL1 enrichment in ubiquitination-associated pathways (Fig. S3A-D). We then treated the cells with MG132 to inhibit proteasomal degradation of ubiquitinated AKT and performed co-immunoprecipitation (Co-IP) analysis, which demonstrated that knockdown of DERL1 significantly suppressed K63 ubiquitination but not K48 ubiquitination of AKT (Fig. 3B-C). This suggests the clear involvement of the K63-linked ubiquitination pathway by which DERL1 modulates AKT activation.

### TRAF6 directly interacts with DERL1 and mediates K63-linked polyubiquitination of AKT

From our immunoprecipitation-mass spectrometry (IP-MS) experiments, we noticed that E3 ligase TRAF6, which is crucial for K63-ubiquitination of AKT, might be one of the candidates having a direct interaction with DERL1 (Supplementary Table 1, Fig. 3D). To establish evidence for this binding partnership between DERL1 and TRAF6, co-immunoprecipitation (Co-IP) and immunoblot assays were performed, which clearly demonstrated the presence of TRAF6 in the precipitate pulled down by the specific antibody against DERL1 (Fig. 3E). Likewise, DERL1 was detected in the precipitate pulled down by the specific antibody against TRAF6 (Fig. 3F). Fluorescence assays too confirmed the colocalization of DERL1 (red) and TRAF6 (green) in ZR-7530 and MDA-MB-231 cells, providing further evidence of their partnership (Fig. 3G). Furthermore, we used Zdock to predict molecular interactions between DERL1 and TRAF6 [33–35]. The docking score between DERL1 and TRAF6 was 2112.101, indicating a stable complex formation. Amino acid residues in the DERL1-TRAF6 interface suggested multiple hydrophobic interactions, further proving the stability of the DERL1/TRAF6 complex (Supplementary Tables 2–3, Fig. 3H, Fig. S3E-G).

**Fig. 4:**
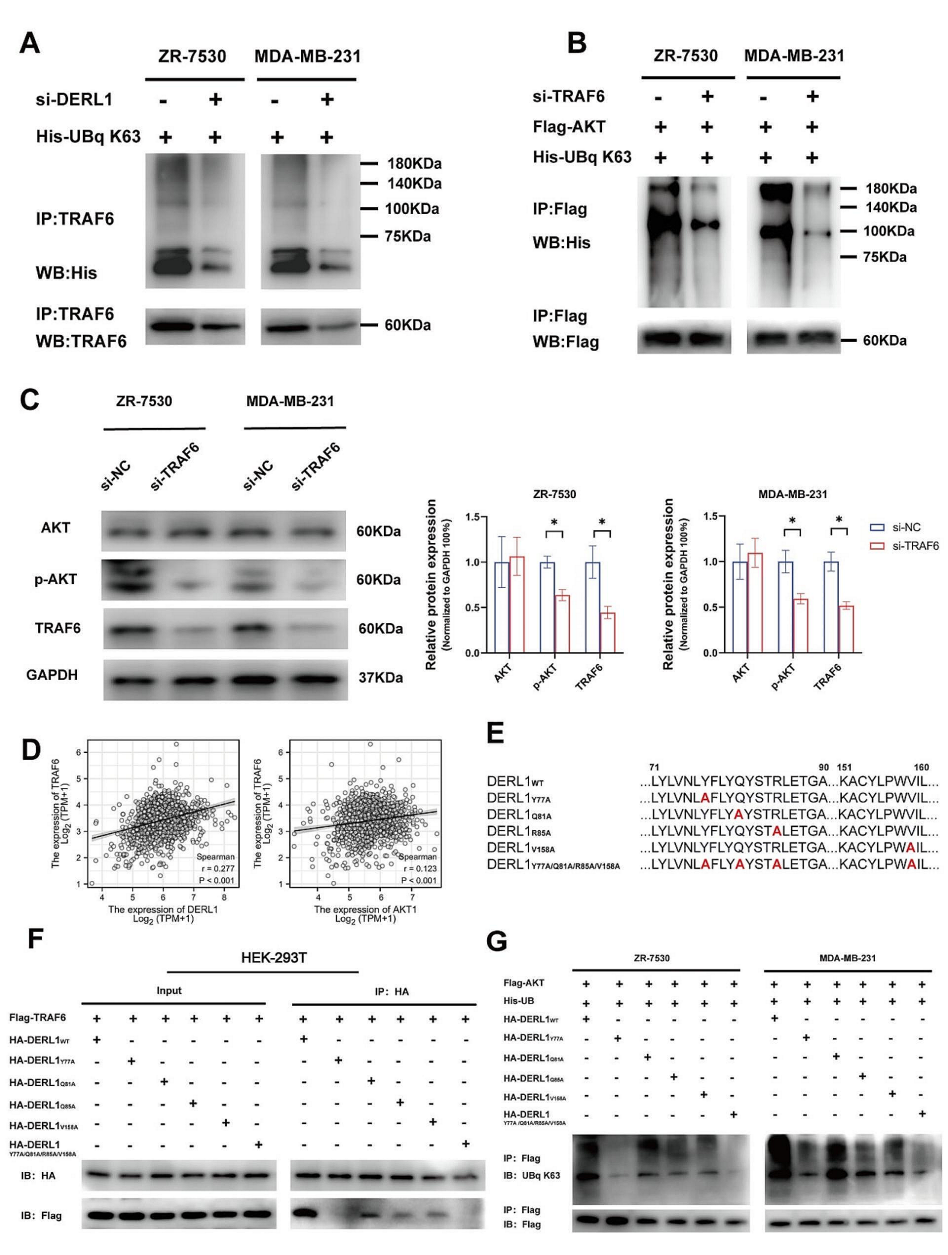
AKT activation by DERL1 is dependent on TRAF6-mediated K63-linked ubiquitination. **A.** ZR-7530 and MDA-MB-231 cells were transfected with si-DERL1 and His-UBq K63, followed by immunoprecipitation with TRAF6 antibody and immunoblotting for K63 ubiquitination. **B.** ZR-7530 and MDA-MB-231 cells were transfected with si-TRAF6, Flag-AKT, and His-UBq K63. Cell lysates were immunoprecipitated with AKT antibody and immunoblotted for K63 ubiquitination. **C.** ZR-7530 and MDA-MB-231 cells were transfected with si-NC or si-TRAF6. Cell lysates were immunoblotted for TRAF6, phospho-AKT, and AKT. **D.** TCGA data demonstrating a positive correlation between TRAF6, DERL1, and AKT in BRCA. **E.** Amino acid mutations in five variants of DERL1. **F.** Expression of Flag-TRAF6 and five HA-tagged DERL1 mutations in HEK-293T cells. Following immunoprecipitation of HA-tagged proteins, TRAF6 was detected by immunoblotting using Flag antibody. **G.** Expression of Flag-AKT, His-UB, and five DERL1 mutations in ZR-7530 and MDA-MB-231 cells. Following immunoprecipitation of Flag-tagged AKT, ubiquitination of AKT was detected by immunoblotting using UBq K63 antibody

We individually knocked down DERL1 in ZR-7530 and MDA-MB-231 cells and observed a corresponding decrease in the levels of K63-ubiquitinated TRAF6 (Fig. 4A). Literature suggests that K63 auto-ubiquitination of TRAF6 is a prerequisite for successive activation of AKT [36]. In cells overexpressing DERL1, TRAF6 silencing inhibited the production of K63-ubiquitinated AKT (Fig. 4B). In addition, the phosphorylation state of AKT was studied via immunoblot after TRAF6 knockdown, suggesting a reduction in p-AKT but not total AKT levels (Fig. 4C). Put together, these findings suggest that TRAF6 mediates with DERL1 in regulating K63-ubiquitinated AKT activation. Furthermore, a look-up at the TCGA database further confirmed a positive correlation between DERL1 and TRAF6 (*r* = 0.277), as well as between TRAF6 and AKT (*r* = 0.123) (Fig. 4D).

We then proceeded with mutation and immunoprecipitation experiments. Using Zdock, we generated predictions of how the TYR-77, GLN-81, ARG-85, and VAL-158 residues of DERL1 would interact with TRAF6. We created four mutant variants by individually substituting the four residues with alanine (Tyr77Ala, Gln81Ala, Arg85Ala, Val158Ala), and a fifth variant by mutating all four residues with alanine simultaneously (Tyr77Ala/Gln81Ala/Arg85Ala/Val158Ala) (Fig. 4E). Subsequently, co-precipitation of HA-DERL1 (WT) and the five mutant variants with Flag-TRAF6 revealed that Tyr77Ala broke the interaction between DERL1 and TRAF6, while Gln81Ala, Arg85Ala, or Val158Ala partially impaired their interaction. Furthermore, a disruption of contact resulted when all four residues were mutated (Fig. 4F).

Co-transfection of Flag-AKT, His-UB, HA-DERL1, or individual DERL1 mutants into ZR-7530 and MDA-MB-231 cell lines separately demonstrated that the expressions of K63-ubiquitinated AKT were reduced to varying extents in the mutant groups compared to the group transfected with HA-DERL1 (WT). Among the mutants, Tyr77Ala and Tyr77Ala/Gln81Ala/Arg85Ala/Val158Ala significantly impaired AKT K63-linked ubiquitination (Fig. 4G).

Collectively, our findings confirm DERL1’s role in the activation of the AKT signaling pathway by interacting with TRAF6 to induce K63-linked ubiquitination of AKT.

### miR-181c-5p directly targets DERL1 and has anti-proliferative and anti-migratory effects on BRCA cells

**Fig. 5:**
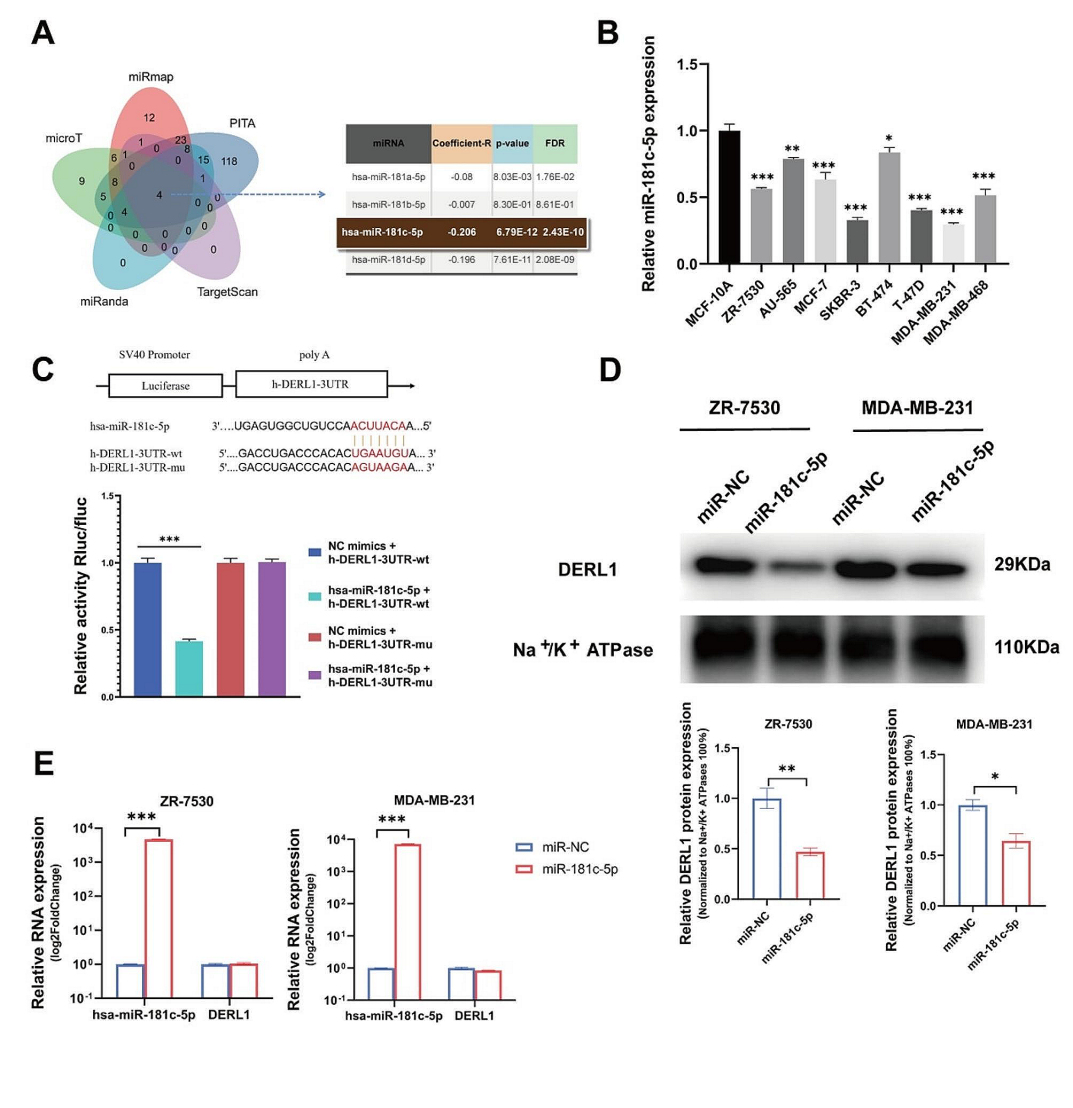
DERL1 is a direct target of miR-181c-5p. **A.** Venn diagrams illustrating the overlap among miRBase, miRanda, PITA, microT, and TargetScan, which are putative miRNAs targeting DERL1. **B.** Relative expression of miR-181c-5p in a BRCA cell line compared to a normal breast cell line. **C.** Renilla luciferase activity, normalized to luciferase, of 293T cells co-transfected with DERL1 WT or mutated mRNA 3’-UTR luciferase reporter constructs together with either miR-181c-5p mimics or miR control mimics. **D.** Overexpression of miR-181c-5p downregulated DERL1 protein expression in ZR-7530 and MDA-MB-231 cells. **E.** Overexpression of miR-181c-5p showing no possibile effect on DERL1 mRNA expression in ZR-7530 and MDA-MB-231 cells. *P < 0.05; **P < 0.01; ***P < 0.001

The transcriptional states of genes are said to be associated with varied biological functions. miRNAs are widely recognized as crucial epigenetic modulators, making them potential targets or predictive markers of diseases [37]. In view of this, we analyzed the potential regulatory miRNAs of DERL1 using programs like PITA, miRmap, microT, miRanda, and TargetScan available in StarBase (https://starbase.sysu.edu.cn/). Venn diagraming revealed four miRNAs at the intersection, namely hsa-miR-181a-5p, hsa-miR-181b-5p, hsa-miR-181c-5p, and hsa-miR-181d-5p. After extracting the correlation coefficients (R) and *P* values between these four miRNAs and DERL1, we found hsa-miR-181c-5p to have exhibited the highest R value (0.206) and the smallest *P* value (6.79E-12) (Fig. 5A). In addition, the expression of miR-181c-5p was lower in BRCA cells compared to MCF-10 A, contrasting the trend of DERL1 (Figs. 1B and 5B). These findings suggest a negative correlation for miR-181c-5p with DERL1.

Dual-luciferase assays revealed significantly reduced luciferase activity for the DERL1 mRNA (WT) with increasing levels of miR-181c-5p, while the mutant counterpart of DERL1 mRNA prevented such reduction, suggesting that miR-181c-5p is able to directly bind with the DERL1 mRNA (Fig. 5C). When examining the pattern by which miR-181c-5p regulates DERL1 in BRCA cells, it became evident that miR-181c-5p reduced the protein levels of DERL1 but did not its mRNA levels (Fig. 5D-E). From these findings, we can infer that miR-181c-5p is capable of suppressing the translation of the DERL1 protein, thus becoming one of the primary therapeutic targets for BRCA.

**Fig. 6:**
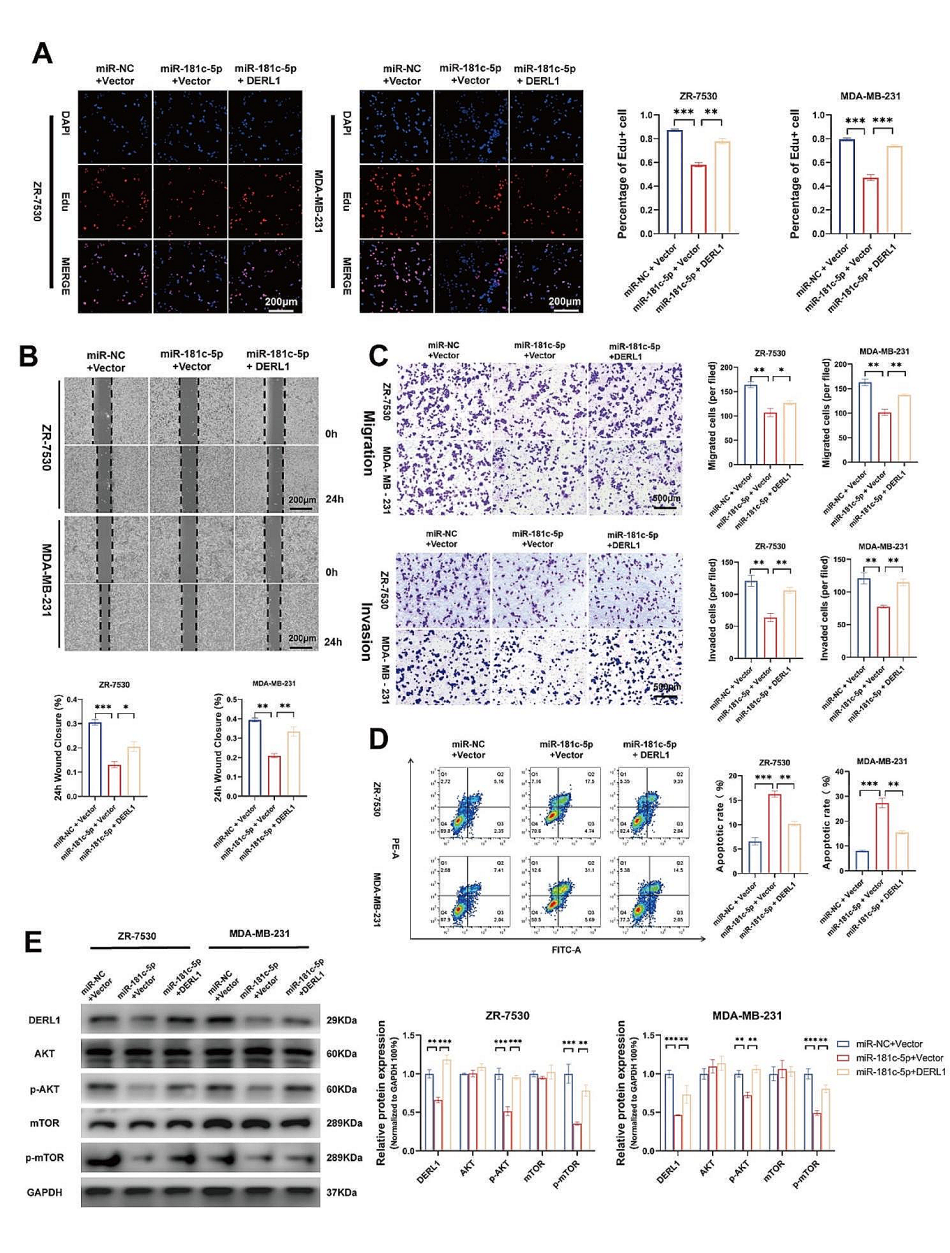
miR-181c-5p exerts an anticancer effect that is mediated by DERL1. **A.** Cell viability of ZR-7530 and MDA-MB-231 was assessed using the EdU assay after the indicated transfections. Scale bar, 200 μm. **B.** Cell migration in ZR-7530 and MDA-MB-231 was measured using a wound-healing assay after the indicated transfections. Scale bar, 200 μm. **C.** Invasive and migratory capacities of ZR-7530 and MDA-MB-231 were evaluated using the Transwell assays after the indicated transfections. Scale bar, 500 μm. **D.** Cell apoptoses in ZR-7530 and MDA-MB-231 were detected using the Annexin V apoptosis detection kit after the indicated transfections. **E.** Overexpression of DERL1 counteracted the suppressive effect of miR-181c-5p on the protein levels in the AKT signaling pathway in ZR-7530 and MDA-MB-231 cells. Data are presented as mean ± SEM (*n* = 3), *P < 0.05; **P < 0.01; ***P < 0.001

We next studied how DERL1 mediates the functioning of miR-181c-5p by employing proliferation, migration, invasion, and apoptosis assays in vitro using ZR-7530 and MDA-MB-231 cells. From its results, we deduce that the upregulation of miR-181c-5p inhibited cell proliferation, invasion, and migration while increasing the proportion of apoptotic cells. However, through restoring DERL1 expression in the presence of miR-181c-5p, the cells’ malignant characteristics and number of apoptotic cells greatly reduced (Fig. 6A-D). With the above findings reaching consistency with immunoblot assays, it can be speculated that miR-181c-5p suppresses p-AKT and p-mTOR production while mediated by DERL1 (Fig. 6E). To add strength to this conclusion, our bioinformatic analyses demonstrated that overall survival (OS), disease-specific survival (DSS), and progression-free survival (PFI) were significantly higher in patients with high miR-181c-5p expression than those with low miR-181c-5p expression (Fig. S3H-J). An inverse relationship between the functions of miR-181c-5p and DERL1 is thus suggested to be at work in BRCA cells.

Summarily, we propose that DERL1 is a direct target of miR-181c-5p and that miR-181c-5p is a potent suppressor of BRCA cells through mediation with DERL1.

## Discussion

Evidence suggesting the role of DERL1 in the progression of various cancers is growing by the day [28, 38–40]. In addition, overexpression of DERL1 has been widely reported for different types of cancers through bioinformatic analyses using the TIMER database [6, 7]. The present study also evidenced overexpression of DERL1 in all subtypes of BRCA through bioinformatic analyses using the TCGA database as well as through experimenting with established BRCA cell lines. The overexpression of DERL1, our study findings suggest, promoted the malignant phenotypes of BRCA cells both in vitro and in vivo. Statistical analyses support our view that overexpression of DERL1 is an independent predictor of poor OS in BRCA patients, corroborating previous studies [30, 39, 41]. Thus, DERL1 may well serve as a potential therapeutic target and prognostic biomarker for BRCA.

A corpus of research has well established that DERL1 promotes BRCA progression by upregulating AKT. After knocking down DERL1 in BRCA cells, our immunoblot assays showed reduced p-AKT and p-mTOR, but not AKT and mTOR levels, imitating previous findings [29, 30, 38]. As GSEA analyses suggested, overexpression of DERL1 could be possibility associated with a ubiquitination-associated signal pathway. Based on a prior study on BRCA cells, the interaction of DERL1 with UBE2C (ubiquitin-conjugating enzyme E2C) facilitated phosphorylation of AKT [30]. As has been widely speculated, the canonical role of DERL1 is essential for the degradation of a subset of misfolded ER proteins [10]. Most recognized E3 ubiquitin ligases, including HRD1, gp78, RNF5, and TMEM129, are all ER membrane-embedded E3 ligases and believed to bind with DERL1 in the ERAD system that is responsible for protein degradation [42, 43]. Using Co-IP assays, we confirmed that DERL1 knockdown in BRCA cells inhibited K63 ubiquitination of AKT, indicating that DERL1 not only has a role in clearing the ER from misfolded proteins but also might be involved in functional modification of proteins.

Having recognized that K63 ubiquitination is a major factor for AKT activation, it must be understood that Nedd4, Skp2, TRAF6, and TRAF4 have been identified as the E3 ligases for AKT K63 ubiquitination [28, 44–47]. We performed IP-MS and found TRAF6 to have significantly interacted with DERL1. Subsequently, both Co-IP and immunoblot assays demonstrated a direct link between DERL1 and TRAF6, in agreement with the results of Zdock analyses. This informs why TRAF6 has been a specific E3 ligase targeted by studies on AKT K63 ubiquitination [23, 27, 28]. In our study, the knockdown of DERL1 reduced auto-ubiquitination of TRAF6. Silencing of TRAF6 in DERL1-overexpressing BRCA cells decreased K63-ubiquitinated AKT as well as p-AKT levels. This was further supported by the positive correlation coefficients between DERL1 and TRAF6, and between TRAF6 and AKT through our TCGA analyses. Using Zdock prediction, we analyzed the impact of TYR-77, GLN-81, ARG-85, and VAL-158 under the premise of DERL1 binding with TRAF6. Co-IP showed that Tyr77Ala and Tyr77Ala/Gln81Ala/Arg85Ala/Val158Ala prevented the interaction between DERL1 and TRAF6, thereby disrupting the production of K63-ubiquitinated AKT. On the other hand, Gln81Ala, Arg85Ala, or Val158Ala partially impaired the binding of DERL1 with TRAF6, causing certain decreases in the expression of K63-ubiquitinated AKT. Collectively our data imply that DERL1 regulates K63-linked ubiquitination of AKT via TRAF6 mediation.

Approximately 60% of protein-coding genes in mammals are regulated by miRNAs. Imagine a single miRNA regulating dozens of mRNAs [48, 49]. In a previous work, miR-30b was found to suppress AKT activation through targeting DERL1 [50]. Based on the prediction results of PITA, miRmap, microT, miRanda, and TargetScan programs in StarBase, we speculate that DERL1 could be a possible target of hsa-miR-181c-5p, in line with our experimental finding of downregulation of miR-181c-5p in BRCA cells. Along the same vein, bioinformatics analysis via the TCGA database too revealed reduced miR-181c-5p levels in all subtypes of BRCA, in turn suggesting that miR-181c-5p might inhibit DERL1 expression. Moreover, the dual-luciferase assay confirmed direct binding of miR-181c-5p with DERL1. Evidently miR-181c-5p overexpression suppressed DERL1 protein translation as well as hindered p-AKT and p-mTOR production. Proliferation, migration, invasion, and apoptosis assays confirmed the inhibitory action of miR-181c-5p on BRCA cells’ malignant phenotypes by targeting DERL1, consistent with literature [21]. When read along with the results of bioinformatic analyses using the TCGA database, the OS, DSS, and PFI are predicted to be better for the miR-181c-5p-high patients than for the miR-181c-5p-low ones. Thus, miR-181c-5p and DERL1-mediated AKT activation hold much promise as therapeutic targets of BRCA.

## Conclusion

**Fig. 7:**
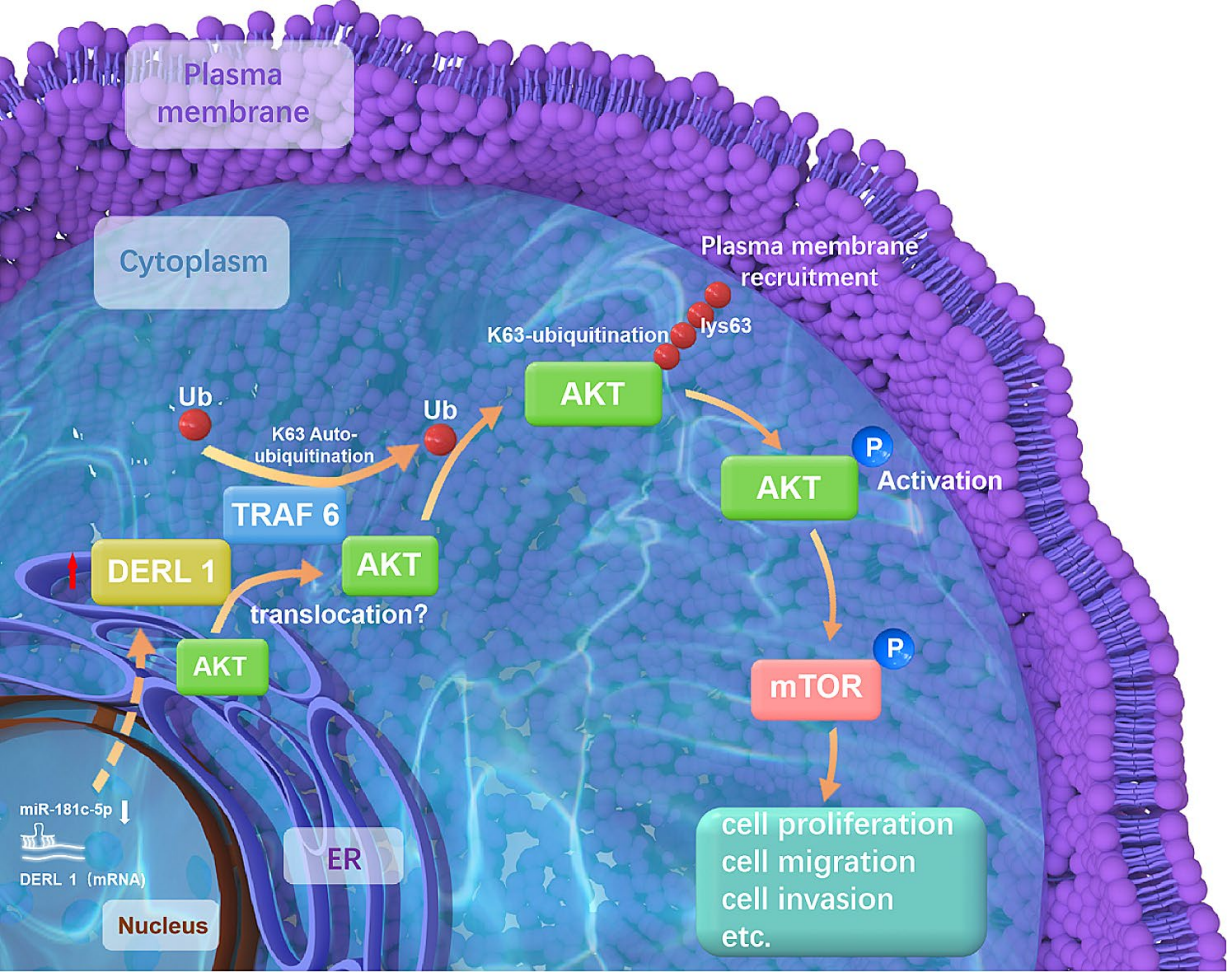
Mediation of miR-181c-5p/DERL1 pathway by TRAF6-linked AKT K63 ubiquitination modulates BRCA progression

The study garnered attention concerning the overexpression of DERL1 in all types of BRCA, suggesting its significance as an oncogenic target and a possible indicator of patients’ OS. DERL1 facilitates AKT activation through K63 ubiquitination. In this study, the interaction between DERL1 and TRAF6 led to enhanced K63-ubiquitinated AKT activation. Under the state of malignancy, DERL1 might sustain translocation of inactive AKT from the ER onto TRAF6 in turn motivating subsequent AKT activation [26, 32] (Fig. 7). Molecular biology is expected to decode the still unknowns regarding the workings of the DERL1/TRAF6/AKT pathways with more precision through ongoing research efforts. Currently AKT-targeted therapy is largely limited since our knowledge base of AKT inhibitors is still in the process of expansion [51]. Our findings might open more doors to the development of therapeutic avenues in BRCA.

### Electronic supplementary material

Below is the link to the electronic supplementary material.

Supplementary Material 1

Supplementary Material 2

Supplementary Material 3

## Data Availability

No datasets were generated or analysed during the current study.

## Abbreviations

BRCA: Breast cancer
IP-MS: Immunoprecipitation-mass spectrum
Co-IP: Co-immunoprecipitation
TCGA: The Cancer Genome Atlas
ER: Endoplasmic reticulum
ERAD: Endoplasmic reticulum-associated degradation
OS: Overall survival
DSS: Disease-specific survival
PFI: Progression-free survival
TPM: Transcripts per million reads
ATCC: American Type Culture Collection
GSEA: Gene set enrichment analysis
RT-qPCR: Reverse-transcription quantitative polymerase chain reaction
EdU: 5-ethynyl-2’-deoxyuridine
HE: Hematoxylin-Eosin
shRNA: Short hairpin RNA
siRNA: Small interfering RNA
PBS: Phosphate-buffered saline
DAPI: 4′,6-Diamidino-2-phenylindole
FBS: Fetal bovine serum
AKT: Protein kinase B
TRAF: Tumor necrosis factor receptor-associated factor
mTOR: Mammalian target of rapamycin
WT: Wild-type
Tyr: Tyrosine
Gln: Glutamine
Arg: Arginine
Val: Valine
Ala: Alanine
