# Supplementary material for: miR-181c-5p/DERL1 pathway controls breast cancer progression mediated by TRAF6-linked K63 ubiquitination of AKT
Source: Cancer Cell Int. 2024 Jun 10;24:204. doi: 10.1186/s12935-024-03395-1 (PMC11165795; doi:10.1186/s12935-024-03395-1)
Supplement: Supplementary file 1 — Supplementary Material 1 [file 12935_2024_3395_MOESM1_ESM.docx]

**Supplementary table. 1** **Identification of DERL1 interacting proteins using IP-MS in ZR7530 and MDA-MB-231 cells**

| **Accession** | **Protein** | **ZR-7530** | **MDA-MB-231** |
| --- | --- | --- | --- |
|  |  | **Coverage [%]** | **Coverage [%]** |
| Q9BUN8 | DERL1 | 66 | 60 |
| Q8TCX0 |  | 56 | 56 |
| A0A1B0GVI3 | KRT10 | 50 | 44 |
| Q562Z4 | ACT | 47 | 47 |
| H6VRF8 | KRT1 | 36 | 33 |
| P35527 | KRT9 | 36 | 33 |
| P08670 | VIM | 35 | 30 |
| P35908 | KRT2 | 33 | 34 |
| A0A0G2JIW1 | HSPA1B | 26 | 27 |
| P12273 | PIP | 26 | 26 |
| Q53G99 |  | 26 | 26 |
| M0R1V7 | UBA52 | 25 | 40 |
| A0A5C2GC48 | IGKV2D-29 | 23 | 23 |
| Q14257 | RCN2 | 22 | 20 |
| Q9Y4K3 | TRAF6 | 21 | 26 |
| E7EUT5 | GAPDH | 21 | 11 |
| Q8NHM4 | PRSS3P2 | 20 | 12 |
| M0QYK5 | U2AF1L4 | 18 | 18 |
| P62633 | CNBP | 15 | 15 |
| F8W6I7 | HNRNPA1 | 14 | 16 |
| H0YHA7 | RPL18 | 14 | 14 |
| A0A5C2GJU6 | IGHV3-7 | 13 | 13 |
| B4DUQ1 |  | 13 | 8 |
| A8K651 |  | 12 | 5 |
| P02533 | KRT14 | 12 | 14 |
| P05141 | SLC25A5 | 12 | 18 |
| P08779 | KRT16 | 12 | 14 |
| P21796 | VDAC1 | 12 | 4 |
| E9PCY7 | HNRNPH1 | 11 | 13 |
| A0A024R326 | RPL29 | 10 | 10 |
| P81605 | DCD | 10 | 20 |
| Q0QEN7 | ATP5B | 10 | 7 |
| A0A7I2V649 | PABPC1 | 8 | 2 |
| E9PKZ0 | RPL8 | 8 | 5 |
| P12236 | SLC25A6 | 8 | 18 |
| A0A7I2V3U2 | NPM1 | 7 | 11 |
| P05388 | RPLP0 | 7 | 23 |
| Q4VB24 | HIST1H1E | 7 | 11 |
| Q53HQ7 |  | 7 | 7 |
| A0A8I5KYP7 | ETFA | 6 | 6 |
| P36578 | RPL4 | 6 | 5 |
| P39023 | RPL3 | 6 | 3 |
| P62241 | RPS8 | 6 | 13 |
| A0A1W2PP74 | GLDC | 5 | 5 |
| B7Z4F6 |  | 5 | 9 |
| B7Z556 |  | 5 | 5 |
| H0Y449 | YBX1 | 5 | 11 |
| B0QYK0 | EWSR1 | 4 | 1 |
| E9PB61 | ALYREF | 4 | 21 |
| P05198 | EIF2S1 | 4 | 4 |
| P35030 | PRSS3 | 4 | 4 |
| A0A024R755 | CALU | 3 | 3 |
| A0A7I2V2S0 | DDX5 | 3 | 3 |
| A0A7I2V464 | NONO | 3 | 9 |
| P04075 | ALDOA | 3 | 8 |
| P53597 | SUCLG1 | 3 | 3 |
| Q53F48 |  | 3 | 3 |
| P25705 | ATP5F1A | 2 | 2 |
| P51991 | HNRNPA3 | 2 | 2 |
| Q5STU3 | DDX39B | 2 | 10 |
| Q9H072 | DKFZp586J151 | 2 | 2 |
| Q9HB00 | DSC1 | 2 | 2 |
| Q13423 | NNT | 1 | 1 |
| Q6MZS5 | DKFZp686A13234 | 1 | 1 |

**Supplementary table. 2 Corresponding residues involved in hydrogen bond formation between DERL1 and TRAF6**

| **DERL1(Green)** | **TRAF6(Grey)** | **bond length** |
| --- | --- | --- |
| TYR-77 | ILE-352 | 3.0 Å |
| GLN-81 | ASN-350 | 2.8 Å |
| ARG-85 | GLU-348 | 2.8 Å |
| VAL-158 | LYS-384 | 3.2 Å |

**Supplementary table. 3 Residues involved in hydrophobic interactions between DERL1 and TRAF6**

| DERL1(Green) | TRAF6(Grey) |
| --- | --- |
| ILE-165、PHE-162、VAL-158、PHE-144、PHE-8、PHE-146、TYR-80、PHE-78、ILE-159、PHE-70、VAL-74、LEU-73、TRP-133、ARG-85、ILE-166 | HIS-421、TYR-418、PRO-385、LEU-494、LEU-435、ILE-354、LEU-422、PRO-423、TYR-387、PHE-426、THR-501、GLY-351、ILE-352、GLY-383、LYS-384、MET-414、LYS-388、TYR-381 |
