# Supplementary material for: miR-181c-5p/DERL1 pathway controls breast cancer progression mediated by TRAF6-linked K63 ubiquitination of AKT
Source: Cancer Cell Int. 2024 Jun 10;24:204. doi: 10.1186/s12935-024-03395-1 (PMC11165795; doi:10.1186/s12935-024-03395-1)
Supplement: Supplementary file 2 — Supplementary Material 2 [file 12935_2024_3395_MOESM2_ESM.docx]

1. **Primers for genes**

Forward primer (5'->3') Reverse primer (5'->3')

DERL1 TCGGACATCGGAGACTGGTT GGCAGTGATTGGCCTCCAAA

AKT1 TCCTCCTCAAGAATGATGGCA GTGCGTTCGATGACAGTGGT

TRAF6 TTCGGAGGGAGGGTTTT GGCTTGTTTGTTTGCATGT

GAPDH GGAGCGAGATCCCTCCAAAAT GGCTGTTGTCATACTTCTCATGG

1. **Primers for micRNAs**

Bulge-LoopTM hsa-miR-181c-5p RT Primer ssD809230185 Guangzhou RiboBio Co., Ltd.

Bulge-LoopTM hsa-miR-181c-5p Forward Primer ssD809230877 Guangzhou RiboBio Co., Ltd.

Bulge-LoopTM miR-Reverse Primer ssD089261711 Guangzhou RiboBio Co., Ltd.

Bulge-LoopTM U6-RT Primer ssD0904071008 Guangzhou RiboBio Co., Ltd.

Bulge-LoopTM miR-Reverse Primer ssD089261711 Guangzhou RiboBio Co., Ltd.

Bulge-LoopTM U6-Forward Primer ssD0904071006 Guangzhou RiboBio Co., Ltd.

Mature hsa-miR-181c-5p Sequence：5-AACAUUCAACCUGUCGGUGAGU-3

hsa-miR-181c-5p Mimic：5-AACAUUCAACCUGUCGGUGAGU-3

1. ACUCACCGACAGGUUGAAUGUU -3

Mimic NC：miR1N0000001-1-5 Guangzhou RiboBio Co., Ltd.

1. **Small interfering RNA and short hairpin RNA sequences**

**Sense (5'->3')** **Antisense (5'->3')**

**siDERL1-1** ACAGAGACAUGAUUGUAUC GAACAGAGACAUGAUUGUAUCAU

**siDERL1-2** CAGAGACAUGAUUGUAUCA AACAGAGACAUGAUUGUAUCAUU

**siDERL1-3** GUAGGAGAGGAGGAGUAUC CAGUAGGAGAGGAGGAGUAUCAG

**si-TRAF6** UACUGCAUCAAUGUCUACAATT UUGUAGACAUUGAUGCAGUATT

**si-NC** UUCUCCGAACGUGUCACGUTT ACGUGACACGUUCGGAGAATT

**sh-DERL1：**

**Top strand:** GATCCGACAGAGACATGATTGTATCTTCAAGAGAGATACAATCATGTCTCTGTTTTTTTG

**Bottom strand:** AATTCAAAAAAACAGAGACATGATTGTATCTCTCTTGAAGATACAATCATGTCTCTGG

**sh-NC：**

**Top strand:**

GATCCGTTCTCCGAACGTGTCACGTAATTCAAGAGATTACGTGACACGTTCGGAGAATTTTTTC

**Bottom strand:**

AATTGAAAAAATTCTCCGAACGTGTCACGTAATCTCTTGAATTACGTGACACGTTCGGAGAACG

1. **Antibodies**

Anti-DERL1 (Abcam, ab176732)

Anti-GAPDH (Proteintech, 60004-1-Ig )

Anti-Na^(+)^/K^(+)^ ATPase 1 (Proteintech, 14418-1-AP )

Anti-AKT (Proteintech, 60203-2-Ig )

Anti-p-AKT (Proteintech, 80455-1-RR )

Anti-mTOR (Cell Signaling Technology, 2983S )

Anti-p-mTOR (Cell Signaling Technology, 2971S )

Anti-TRAF6 (Abcam, ab137452)

Anti-Ubiquitin linkage-specific K48 (Abcam, ab140601)

Anti-Ubiquitin linkage-specific K63 (Abcam, ab179434)
