# Supplementary material for: miR-181c-5p/DERL1 pathway controls breast cancer progression mediated by TRAF6-linked K63 ubiquitination of AKT
Source: Cancer Cell Int. 2024 Jun 10;24:204. doi: 10.1186/s12935-024-03395-1 (PMC11165795; doi:10.1186/s12935-024-03395-1)
Supplement: Supplementary file 3 — Supplementary Material 3 [file 12935_2024_3395_MOESM3_ESM.docx]

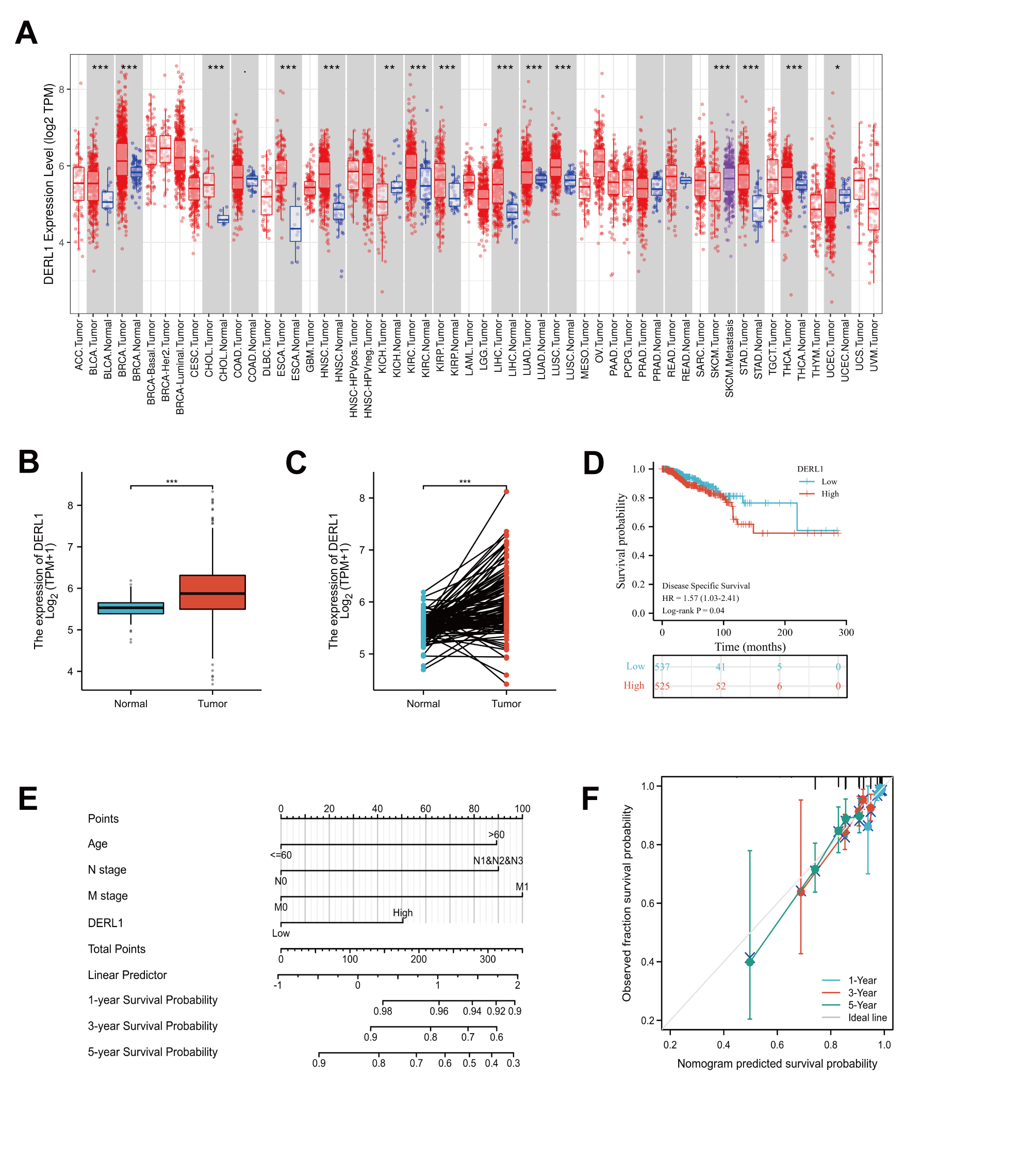
**Supplementary Fig. 1**

**Expression status of DERL1 in BRCA and its prognostic value.**

**A.** Comparison of DERL1 expression levels in different cancers using data from the TCGA database.

**B-C.** Differential expression levels of DERL1 in BRCA.

**D.** Disease-specific survival (DSS) curves comparing DERL1-high and DERL1-low patients with BRCA using TCGA data.

**E.** Nomogram for predicting the probability of 1-, 3-, and 5-year overall survival (OS) in BRCA patients.

**F.** Calibration plots of the nomogram for predicting the probability of OS at 1, 3, and 5 years.

**p* < 0.05; ***p* < 0.01; ****p* < 0.001.


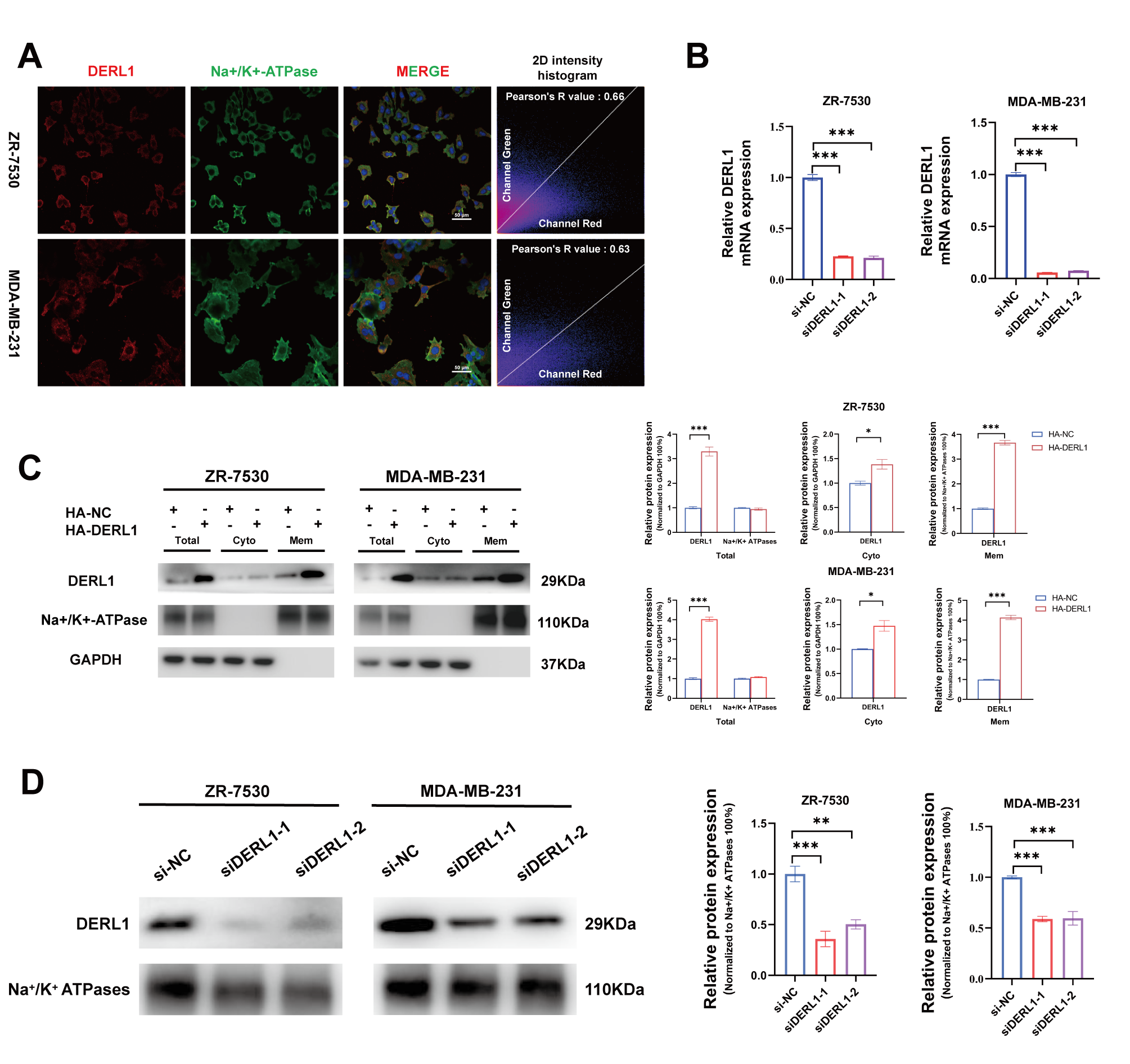


**Supplementary Fig. 2**

**Localization of DERL1 in BRCA cells and interference DERL1 expression in cells.**

1. Immunofluorescence staining of ZR-7530 and MDA-MB-231 cells using anti-DERL1 monoclonal antibody (Red) and anti-Na+/K+-ATPase polyclonal antibody (Green). Nuclei were stained with DAPI. Images were captured at a magnification × 40 with 10 × digital zoom, and scale bars represent 50 µm.

**B.** The cellular membrane and cytoplasmic fractions were isolated from cells, and the protein levels of DERL1 in each fraction were determined using Western blot analysis. Na+/K+-ATPase or GAPDH was used as a loading control for membrane protein or total protein, respectively.

**C.** Downregulation of DERL1 mRNA expression in BRCA cell lines by transfection with siDERL1-1 or siDERL1-2.

**D.** Downregulation of DERL1 protein expression in BRCA cell lines by transfection with siDERL1-1 or siDERL1-2.

Data are presented as mean ± SEM, **p* < 0.05; ***p* < 0.01; ****p* < 0.001.


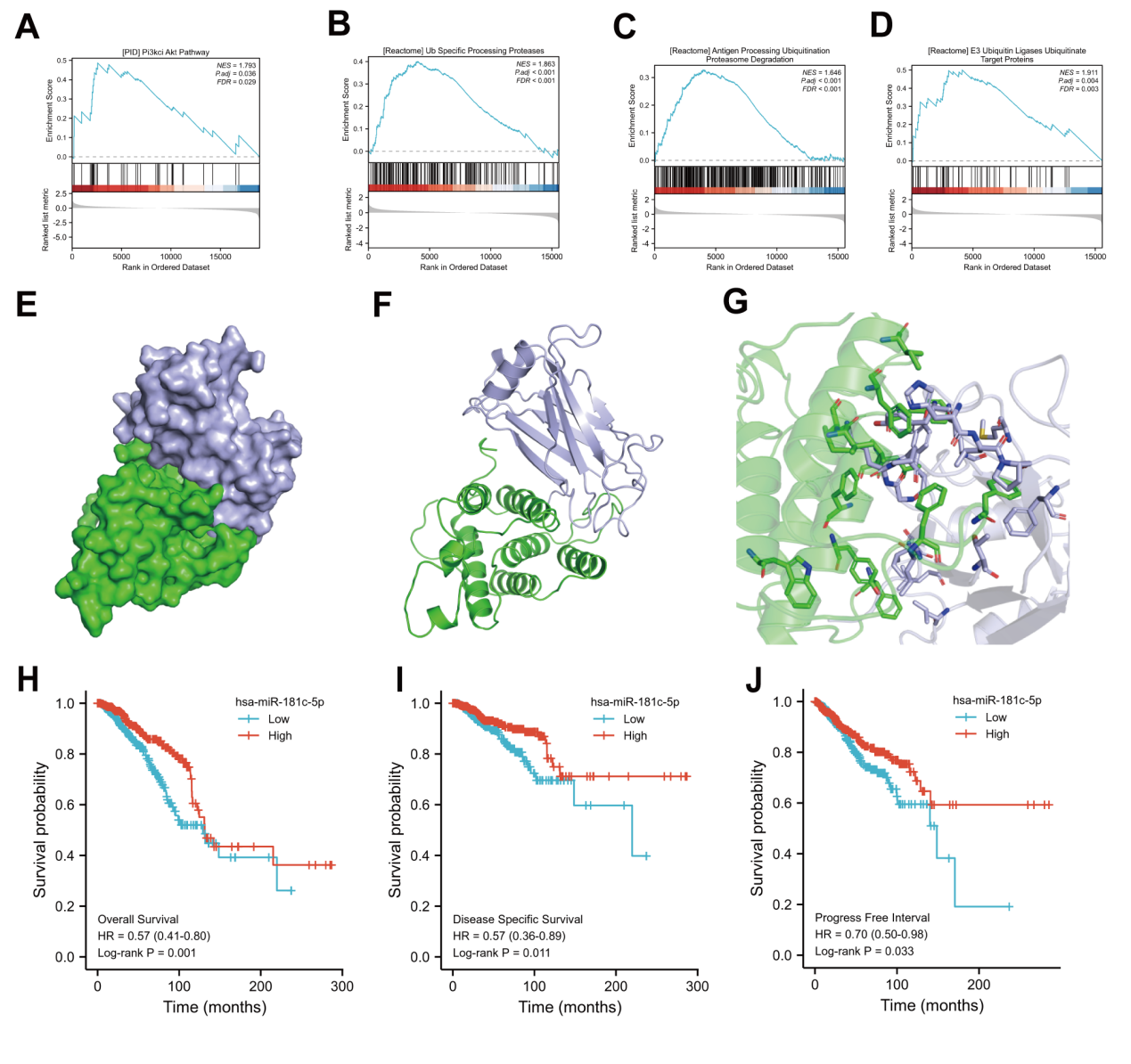


**Supplementary Fig. 3**

**Mechanistic analyses for DERL1, and prognosis prediction of miR-181c-5p.**

**A-D.** Enrichment plots obtained from gene set enrichment analysis (GSEA) reveal differential enrichment of several ubiquitination-associated pathways in DERL1-high BRCA cells.

**E-F.** Molecular docking was performed to establish the binding model of DERL1 (PDB ID: 7CZB) and TRAF6 (PDB ID: 1LB6). The complex of DERL1 (Green) and TRAF6 (Grey) is represented in a cartoon format.

**G.** Amino acid residues of DERL1 (Green) and TRAF6 (Grey) form hydrophobic interactions, contributing to the hydrophobic surface of the complex.

**H-J.** Survival curves of overall survival (OS), disease-specific survival (DSS), and progression-free interval (PFI) between miR-181c-5p-high and -low patients with BRCA in TCGA data.

NES: Normalized Enrichment Score; *p*.adj: Adjusted *p* value; FDR: False Discovery Rate.
